# Epidemiology of childhood bone and joint disease during the COVID-19 pandemic in New Zealand

**DOI:** 10.1007/s15010-024-02356-0

**Published:** 2024-08-02

**Authors:** Sarah Hunter, Elsie Brown, Haemish Crawford, Cameron Grant

**Affiliations:** 1 Faculty of Medical and Health Sciences, University of Auckland, Auckland, New Zealand; 2 Paediatric Orthopaedic Department, Starship Hospital, Grafton Road, Zealand, New Zealand; 3 Department of Child and Youth Health, Starship Hospital, Zealand, New Zealand

## Abstract

**Purpose:**

It is unknown whether social distancing impacts frequency of presentation and severity of childhood bone and joint infection (BJI). In New Zealand, the COVID-19 disease elimination strategy involved strict social isolation policies spanning March 2020-September 2022. Examination of this period may provide insight around risk factors for BJI.

**Methods:**

A retrospective review of all patients < 16 years with presumed acute haematogenous osteomyelitis (AHO) or septic arthritis (SA) treated in the Auckland region was performed between 2018 and 2023. Frequency and severity of presentations has been examined before, during, and after periods of social restriction. Severe cases included those with intensive care admission, recurrent infection, or multiple surgeries. Pre-hospital experience, length of stay, and disease outcomes have also been assessed.

**Results:**

A total of 563 cases met inclusion criteria. Compared to the pre-pandemic period, monthly case averages reduced between April 2020 to September 2022 (10.1 vs. 7.9 cases/month, *p* = 0.008). Separating cases by causative microbiology shows a statistically significant drop in culture negative and *Kingella kingae* mediated BJI cases (4.2 vs. 2.9 cases/month, *p* = 0.006) but not for cases secondary to *Staphylococcus aureus* and *Streptococcus pyogenes* (4.2 vs. 3.9 cases/month, *p* = 0.6). The frequency of severe disease reduced during this period (5.6 vs. 4.1 cases/month, *p* = 0.01) together with lower rates of recurrent infection (9% vs. 4%, *p* = 0.03).

**Conclusion:**

The COVID-19 management strategy in New Zealand utilised strict social isolation, mask wearing, and hand hygiene measures to control disease spread between 2020 and 2022. These measures coincided with reduction in frequency and severity of presentations for childhood BJI.

## Introduction

In March 2020 New Zealand responded to the COVID-19 pandemic by initiating one of the strictest lockdowns worldwide [1]. Known as going ‘hard’ and ‘early’, the goal was to eliminate community spread of the virus. Border closures, hygiene advice, physical distancing, and masking wearing were utilised alongside tracking and tracing active cases [2]. Community restrictions did not formally lift for more than two and a half years, in September 2022. [3]

The unique environment created in response to COVID-19 presents an opportunity to study the effects of social isolation on unrelated diseases. A modification in the transmission of traditional respiratory pathogens has already been noted, with reductions in influenza and respiratory syncytial virus infections during pandemic lockdowns [4]. Perhaps more surprisingly, Group A streptococcus (GAS) infections fell in response to lockdown measures but then rose higher than pre-pandemic levels with signs of increased virulence in late 2022 [4]. It is unclear whether this represents an ‘immunity debt’ among post-lockdown patients or whether GAS infections are concomitant with other respiratory viral illnesses [5]. 

New Zealand is established as having one of the highest rates of childhood bone and joint infection (BJI) in the world, with incidence > 40 cases/100,000/year. [6] Cases of acute haematogenous osteomyelitis (AHO) and septic arthritis (SA) are predominantly mediated by *Staphylococcus aureus.* Inequitable disease burden is seen among indigenous Māori and Pacific children, who experience rates of disease 2-3x times higher than their peers [7]. Case severity ranges from isolated disease treated with intravenous antibiotics to those with septic shock and disseminated infection requiring multiple operations and admission to intensive care [8]. 

Reasons for high levels of invasive *S. aureus* disease in NZ are not fully understood. It is hypothesised that socioeconomic deprivation and community overcrowding play a part [9]. Prior to lockdown measures, social distancing was considered a non-modifiable variable when examining childhood BJI.

Epidemiological analysis of childhood BJI during the period of COVID-19 restrictions will help delineate the effects of social distancing and increased hygiene measures on rates of disease. Compared to other regions where lockdowns ceased earlier or were less austere, the prolonged and stringent measures undertaken by the NZ government may have created more profound epidemiologic change [10]. 

The object of this study is to examine the frequency and severity of presentations of SA and AHO immediately before, during, and after the COVID-19 restrictions in New Zealand. Subgroup analysis will be conducted by pathogen type, with attention to pre-hospital care and disease outcomes.

## Methods

Health and Disability Ethics Committee (HDEC) approval was obtained for this work together with institutional review board approval (reference: **19/NTA/46).** This cohort study is a retrospective analysis of all cases of suspected osteomyelitis and septic arthritis managed in the Auckland region between 2018 and 2023. Children from newborn to age 15-years were included.

Comprehensive review of electronic clinical records was conducted. Data was collected on patient demographics, disease type, and microbiology. Acute haematogenous Osteomyelitis (AHO) was defined using magnetic resonance imaging (MRI) or computed tomography (CT) and/or positive intra-operative culture or bone biopsy. We defined septic arthritis (SA) based on intraoperative culture results, culture results from aspirate, or operative appearance consistent with infection. Cases of chronic infection, post-viral or reactive arthritis, post-operative infection, cases associated with significant malignancy, or patients with insufficient clinical data for analysis have been excluded. Children with a primary diagnosis of osteomyelitis with contiguous infection of the adjacent joint or muscle were included.

Microbiological samples were used from either joint aspirate, blood culture, or intra-operative specimen. Standard agar plate culture was used for synovial fluid aspirate, positive result defined by positive gram stain, cell count > 50,000mm^3^, growth of pathogen on culture, or intra-operative findings consistent with infection. 16sPCR is also utilised in the Auckland region for selected cases.

Cases were considered to represent severe and complicated disease if the child met specific criteria. This criterion includes admission to a paediatric intensive care unit (PICU), multiple surgeries to control infection, recurrent infection and/or re-admission for further debridement, and children with multifocal sepsis.

Pre-hospital information has been collected including primary care attendance before hospital, any initial misdiagnosis, and duration of symptoms before starting treatment. A patient is considered to be misdiagnosed if the first diagnosis was not AHO or SA. For example, a case would be classified as having initial misdiagnosis if a child with extremity pain was discharged with a diagnosis of transient synovitis, re-presents, and is subsequently diagnosed with infection. Length of stay, number of outpatient clinic visits, and rates of treatment failure have been calculated. General practitioner referral records were reviewed to document recent viral illnesses.

Monthly case numbers have been compared in the period before, during, and after COVID-19 restrictions. Case numbers were divided into subgroups by causative pathogen and severity of illness.

The timeframes for analysis are defined by the periods of COVID-19 mandates initiated by the NZ government. ‘Pre-pandemic’ cases analysed in this study are those presenting between January 2018 and March 2020 [3]. A period of social isolation commenced toward the end of March 2020 and general community restrictions were not lifted until September 2022. The strictest lockdowns were enforced between March-May 2020 and August-December 2021. During these strict lockdowns, educational facilities, daycares, and public venues were generally closed and non-urgent elective surgeries were canceled. A list of regulations for early childhood education centres has been provided in Table 1. This is not exhaustive, but provides an example from public health guidelines published in September 2020 [11]. Additional legislation was passed to facilitate enforcement of contact tracing and other public health regulations [12]. Outside of strict lockdowns, there were still restrictions in the community including mask wearing, limits on gatherings, and hand hygiene rules [1]. ‘Post-pandemic’ cases are defined between October 2022 and August 2023.

**Table 1:** Examples of COVID-19 restrictions in Education and Childcare facilities

	Examples of Restrictions and Measures to Reduce COVID-19 Spread in Schools and Early Childhood Centres[1]		
	General Periods between lockdowns	Most severe restrictions (e.g. ‘Level 3 or Level 4’)	
Attendance	Centres able to open to capacity if they meet public health requirements	All schools and early learning centres closed at high alert. In moderate alerts, families advised to keep children at home. Limited daycare facilities (e.g. for essential workers) allowed to open if physical space of 3sqm/child indoors and 5sqm/child outdoors.	
Distancing	Practical guide of 1 m between adults in public spaces and no large gatherings > 100 people*	No social contact outside of immediate family group/bubble. Children in groups of ten in daycare spaces*	
Indoor Toys and resources	All toys and resources can be used	Resources divided amongst groups of children (e.g. one group of ten allowed to use a set of books per day). Toys that cannot be cleaned every day are to be put away.	
Outdoor Toys and resources	All toys and resources can be used	Outdoor play in one group of children (one group of ten) at a time, no sandpits to be used. No excursions.	
Cleaning	Disinfect and clean all surfaces daily	Disinfect and clean all surfaces daily. No food sharing. Meal breaks staggered.	
Contacts	Contact tracing registers in place for all staff, children, and visitors with use of QR poster	Contact tracing registers in place for all staff, children, and visitors with use of QR poster	
Sickness	Closure of services with identified cases for minimum of 72 h. Contact tracing performed by Ministry of Health with self-isolation for contacts.	Closure of services with identified cases for minimum of 72 h. Contact tracing performed by Ministry of Health with self-isolation for contacts.	

1. Ministry of Education. Health and safety in licensed early learning services and kōhanga reo for COVID-19 [Internet]. Guidance for Alert Levels 1–4. 2020 [cited 2024 Jul 10]. Available from: https://www.education.govt.nz/assets/Uploads/Health-and-safety-for-COVID-19-Licensed-Early-Learning-Services-Alert-Levels-1-4.pdf

*Mask wearing in schools was enforced from age 8 upwards but not for younger children.

## Results

An initial 994 cases were identified by clinical coding. Of these, 563 met criteria for acute BJI (Table 1). The majority had a primary diagnosis of AHO (77%). *Staphylococcus aureus* was the most likely causative pathogen followed by culture negative cases. *Kingella kingae* was identified in 23 samples using PCR (7.4% of all positive samples).

Complicated and/or severe illness was seen in 58% of cases; this was most likely due to readmission for treatment failure (*n* = 106, 19%), multifocal sepsis (*n* = 90, 16%), and children requiring multiple operations to control disease (*n* = 85, 15%). Of note, 39 children (7%) were managed in intensive care. A total of 30 children (5%) developed chronic infection.

**Table 2:** Cohort characteristics

Cohort Characteristics all cases of AHO and SA, 2018–2023, *N* = 563		
Electronic Case Screening with reasons for Exclusion		
Total number of Records Screened:	**994**	
Number meeting criteria:	**563**	
Excluded:	**431**	
Soft tissue abscess/cellulitis	12	
Chronic Infection	29	
Secondary to pressure sore/ulceration	2	
Secondary to significant medical condition e.g. disseminated malignancy	29	
Secondary to #, operative complication, or penetrating injury	56	
Chronic Recurrent Multifocal Osteomyelitis	33	
Re-admission for same condition	161	
Alternative final diagnosis	74	
Location of exclusion e.g. frontal bone, sinus	31	
Tuberculosis	4	
Basic Demographics	Number	**%**
Disease Type		
Acute Haematogenous Osteomyelitis (AHO)	427	76%
Septic arthritis (SA)	134	24%
Severe or Complicated Disease Course (*N* = 328)		
Intensive Care Admission	39	7%
Multifocal sepsis	90	16%
> 1 Surgery to control infection	85	15%
Chronic infection	30	5%
Readmission for treatment failure or further surgery	106	19%
Causative Pathogen		
*Staphylococcus aureus*	236	42%
*Streptococcus pyogenes*	30	5%
*Kingella kingae*	23	4%
Culture negative	214	38%

**Fig. 1:**
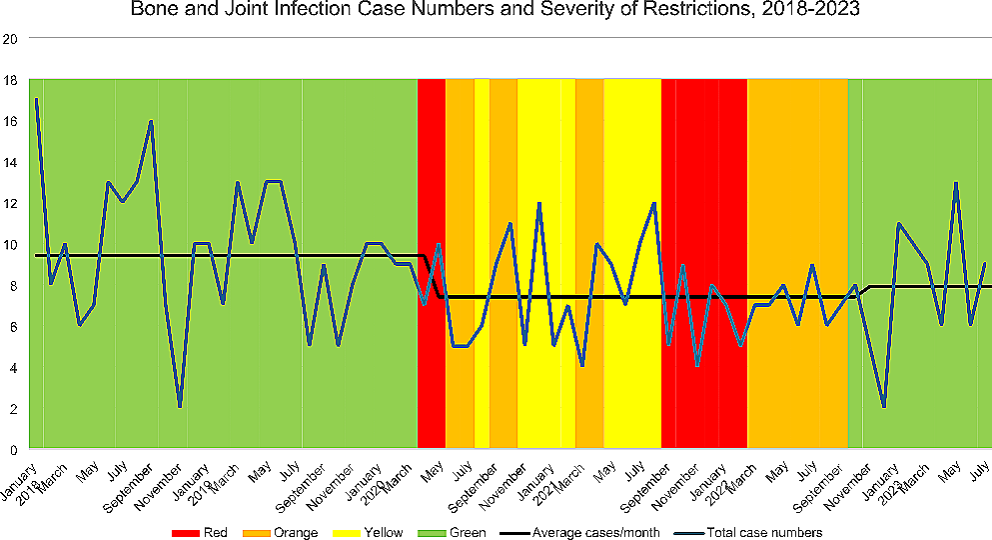
Total number of cases by severity of restrictions, 2018–2023. Green: No community restrictions (NB: mask wearing in healthcare settings such as hospitals remained in place until August 2023). Red/Orange-Periods of highly restricted social interaction. Significant dates as follows: 1) March 20th -June 8th 2020 alert levels between 2-4 Nationally. 2) August 12th - October 7th 2020 alert levels between 2-3 in Auckland region. 3) February 14th -March 12th 2021 alert levels between 2-3 in the Auckland region. 4) August 17th – Dec 2nd 2021 alert levels between 2-4 in Auckland region after which alert system abolished, traffic light setting commenced. 5) Dec 15th 2021 regional Auckland border lifted. 6) Sept 12th 2022 community restrictions ended. Yellow: Periods of mildly restricted social interaction, mask wearing, vaccine mandates and hand hygiene. Black line: Mean number of cases per month before, during, and after restrictions. Blue line: Total number of cases per month

Between January 2018 and March 2020, prior to the COVID-19 pandemic, there were an average 10.1 cases/month (+/-4). During the period of COVID-19 restrictions, ending September 2022, case numbers dropped significantly to 7.9/month (+/-2.5) (*p* = 0.006) (Table 3; Fig. 1). Proportion of children admitted aged between 6 and 48 months appeared to reduce but did not reach statistical significance (27% vs. 34%, *p* = 0.16).

**Table 3:** Pre-hospital care, morbidity, and outcomes

Pre-hospital care, Morbidity, and Follow-up Pre and Post COVID-19 Restrictions 2018–2023							
Variable	Pre-COVID-19 Restrictions		During COVID-19 restrictions		After COVID-19 Restrictions		CHI2/TTEST (pre vs. during)
	N	%/SD	N	%/CI	N	%/SD	
Total Number of Cases	261		223		79		
Population based incidence for those </=14 Auckland catchment from 2018 NZ Census[1] (95% CI)	22.64	18.68–26.9)	17.1	(14.13–21.14)	17.45	(14.13–21.14)	0.06
Average cases/month	10.1	+/-4	7.9	+/-2.5	7.9	+/-3.2	0.006
Serious illness cases/month	5.6	+/-2.6	4.1	+/-1.5	4.5	+/-1.5	0.006
Cases in children 6–48 months of age	89	34%	61	27%	32	41%	0.13
S. aureus/S. pyogenes positive cases/month	4.2	+/-1.6	3.8	+/-1.7	4.3	+/-2.1	0.4
Culture negative/K. kingae cases/month	4.2	+/-2.5	2.9	+/-1.7	2.9	+/-1.7	0.04
Sought primary care prior to hospital attendance	117	45%	143	64%	50	63%	< 0.05
Mean duration of symptoms in days prior to treatment	4.9	+/-4.7	5.9	+/-6.5	5.9	+/-5.6	0.03
Initial misdiagnosis	98	38%	87	39%	33	42%	0.7
Median Length of stay in days	10.9	+/-11.1	11.1	+/-14.8	10.2	+/-11.1	0.8
Median number outpatient clinics	3	+/-3.6	2	+/-4.5	2	+/-2.5	0.5
Number of children experiencing recurrent or chronic infection	23	9%	8	4%	4	5%	0.03

Legend: Pre COVID-19 Restrictions defined as period between January 2018-March 2020. During COVID-19 Restrictions from end of March 2020-September 2022. After COVID-19 Restrictions between October 2022-August 2023.

S. Aureus = Staphylococcus aureus, S. pyogenes = Streptococcus pyogenes, K. kingae = Kingella kingae

1. NZ S. 2018 Census and Dwelling Counts [Internet]. Stats NZ Tauranga Aotearoa. 2018 [cited 2019 Nov 26]. Available from: https://figure.nz/chart/rsD7EYYeyZ4dqL0Z

**Fig. 2:**
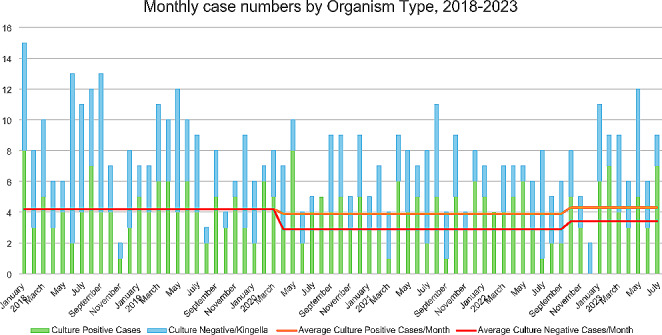
Monthly Case Numbers Separated by Causative Organism, 2018–2023

Cases with microbiologic samples positive for *S. aureus* or *S. pyogenes* did not reduce significantly during this time (4.2 vs. 3.8, *p* = 0.4). Conversely, rates of culture negative BJI and cases of *K. kingae* showed statistically significant reduction (4.2 vs. 2.9, *p* = 0.04) (Fig. 2). These were combined in analysis as *K. kingae* is the most common cause of culture-negative septic arthritis.

**Fig. 3:**
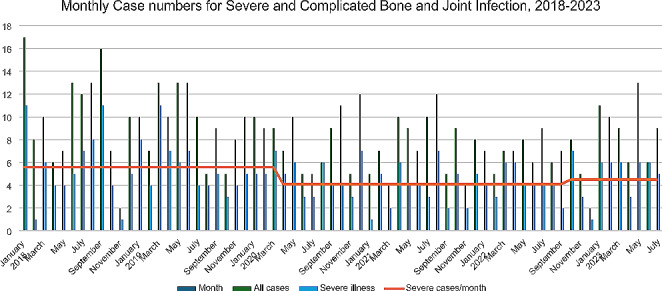
Monthly Cases of Bone and Joint Infection by Disease Severity, 2018–2023

With respect to disease severity, there were 5.6 cases/ month (+/- 2.6) classed as severe or complicated prior to COVID-19 restrictions. Frequency of severe illness underwent a statistically significant reduction during the COVID-19 period, dropping to 4.1 cases/month (+/- 1.5, *p* = 0.01) (Fig. 3).

Examining pre-hospital care, a greater proportion of families presented to primary care during the pandemic period compared to the year prior (45% vs. 64%, p = < 0.05) and the duration of symptoms prior to treatment initiation was longer (5.9 vs. 4.9 days, *p* = 0.03). The rate of misdiagnosis was unchanged (38% vs. 39%, *p* = 0.7). Considering the entire cohort, almost half of children reported a recent viral illness before being diagnosed with BJI (46%).

Median length of stay and number of outpatient clinics were not statistically different. However, the number of children who experienced recurrent or chronic infection reduced significantly from pre-pandemic period (9% vs. 4%, *p* = 0.03).

## Discussion

Restrictions introduced during the COVID-19 pandemic significantly reduced rates of childhood osteomyelitis and septic arthritis in the Auckland region. The effects of this continue to be evident as the average number of cases per month has not returned to pre-pandemic levels.

Severity of disease was also lowered during pandemic restrictions. There was a statistically significant reduction in the monthly number of children with multifocal sepsis, PICU admissions, and those requiring multiple operations. Long-term outcomes potentially improved, with recurrence rate reduced by more than half during the COVID-19 period.

Culture negative cases were disproportionately affected compared to cases secondary to *S. aureus* and *S. pyogenes*. Whilst both appeared to fall during the pandemic, only culture negative cases or those mediated by *K. kingae* achieved statistically significant reduction.

Primary healthcare utilisation increased during the COVID-19 period. This may have contributed to the longer average duration of symptoms prior to treatment initiation, which rose during the pandemic period by approximately one day.

There are several possible reasons for the reduced rate and severity of disease noted by our research. Firstly, improved hygiene and mask wearing may have contributed to the reduction in culture negative cases. Recent studies have hypothesised that the majority of culture negative BJI may be attributable to *K. kingae*, particularly in younger children. This organism is transmitted via respiratory secretions and saliva. Like other respiratory pathogens, the transmission is effectively lowered by mask wearing. [13, 14] In settings where *K. kingae* has colonised the oropharynx, viral infections of the upper respiratory tract damage the mucosal layer and facilitate *K. kingae* penetration into the bloodstream and skeletal system. Reduced incidence of respiratory viruses may therefore reduce burden of disease secondary to this pathogen. [15]

Secondly, a reduction in culture-positive disease secondary to *S. aureus* or *S. pyogenes* may be in part a direct consequence of reduced skin contact in this period of social isolation. Family gatherings, reduced school and day care attendance, and public social distancing all occurred during the study period. [16]

However, another proposed aetiology of infection suggests that GAS or Staphylococcal infections can be concomitant with respiratory viruses [4, 5]. Importantly, this means that a viral infection may play a role in converting colonisation to invasive disease. It should be reiterated that 46% of children in this study reported a viral infection in the weeks leading up to their admission with invasive BJI. Respiratory virus transmission, particularly influenza and rhinovirus, reduced significantly in NZ during the pandemic [17]. Future studies are required to better characterise the relationship between respiratory pathogens and *S. aureus* mediated osteomyelitis and septic arthritis.

The two major strengths of this research include unique study setting and thorough evaluation of individual-level data. Compared to other high-income countries, NZ experienced negative excess mortality. The elimination strategy was austere, effective, and relied on radical changes in social structure. For the purposes of this research, it can be determined that limiting gatherings in schools, day care facilities, and public venues lowered childhood BJI admissions from 10.1 to 7.9 cases/month [1, 2, 10]. 

Secondly, the evaluation of individual level data has not yet been performed for childhood BJI in the setting of COVID-19. Other reports of reduced infection rates have been limited by simple reporting of diagnosis related group information. This does not allow for an assessment of pre-hospital care, microbiology, or treatment outcomes [18–21]. In particular, recent viral infections were more reliably documented in general practitioner records.

This study is limited by the quality of recorded electronic case information. It should be noted that 16s PCR, although available in our region, is not routinely performed in every case, leading to potential underreporting of *K. kingae*. [15, 22]

In conclusion, the COVID-19 pandemic in New Zealand provides a unique opportunity to evaluate risk factors for childhood bone and joint disease. Measures undertaken to eliminate spread of COVID-19 coincided with a significant reduction in frequency and severity of presentations for childhood BJI.

## Data Availability

No datasets were generated or analysed during the current study.
